# Metastasis of prostate cancer and melanoma cells in a preclinical *in vivo* mouse model is enhanced by L-plastin expression and phosphorylation

**DOI:** 10.1186/1476-4598-13-10

**Published:** 2014-01-18

**Authors:** Selina M Riplinger, Guido H Wabnitz, Henning Kirchgessner, Beate Jahraus, Felix Lasitschka, Bianca Schulte, Gabri van der Pluijm, Geertje van der Horst, Günter J Hämmerling, Inaam Nakchbandi, Yvonne Samstag

**Affiliations:** 1 Institute for Immunology, Ruprecht-Karls-University, Heidelberg, Germany; 2 Department of Pathology, Ruprecht-Karls-University, Heidelberg, Germany; 3 Department of Urology, Leiden University Medical Center, Leiden, The Netherlands; 4 Division of Molecular Immunology, German Cancer Research Center DKFZ, Heidelberg, Germany; 5 Translational Medicine, Max-Planck Institute for Biochemistry, Martinsried, Germany

**Keywords:** L-plastin, Actin cytoskeleton, Metastasis, Prostate cancer, Melanoma

## Abstract

**Background:**

Tumor cell migration and metastasis require dynamic rearrangements of the actin cytoskeleton. Interestingly, the F-actin cross-linking and stabilizing protein L-plastin, originally described as a leukocyte specific protein, is aberrantly expressed in several non-hematopoietic malignant tumors. Therefore, it has been discussed as a tumor marker. However, systematic *in vivo* analyses of the functional relevance of L-plastin for tumor cell metastasis were so far lacking.

**Methods:**

We investigated the relevance of L-plastin expression and phosphorylation by ectopical expression of L-plastin in human melanoma cells (MV3) and knock-down of endogenous L-plastin in prostate cancer (PC3M). The growth and metastatic potential of tumor cells expressing no L-plastin, phosphorylatable or non-phosphorylatable L-plastin was analyzed in a preclinical mouse model after subcutaneous and intracardial injection of the tumor cells.

**Results:**

Knock-down of endogenous L-plastin in human prostate carcinoma cells led to reduced tumor cell growth and metastasis. Vice versa, and in line with these findings, ectopic expression of L-plastin in L-plastin negative melanoma cells significantly increased the number of metastases. Strikingly, the metastasis promoting effect of L-plastin was not observed if a non-phosphorylatable L-plastin mutant was expressed.

**Conclusions:**

Our data provide the first *in vivo* evidence that expression of L-plastin promotes tumor metastasis and, importantly, that this effect depends on an additionally required phosphorylation of L-plastin. In conclusion, these findings imply that for determining the importance of tumor-associated proteins like L-plastin a characterization of posttranslational modifications is indispensable.

## Background

Development of metastasis causes the most serious clinical consequences of cancer and is responsible for over 90% of cancer related deaths [1]. The process of metastasis is complex. It involves release of tumor cells from the primary tumor, intravasation, dissemination within vessels, adhesion to vessel walls, extravasation as well as invasion and migration into the tissue of distant organs in order to colonize and form metastases. These steps require a high motility of cancer cells, which is mediated by modulation of the cellular cytoskeleton (for review see [2,3]). Recent studies indicate cytoskeleton binding proteins as important players in tumor metastasis [4,5] (for review see [6]), particularly by their capability to bind to and regulate integrin molecules [7]. Therefore, such molecules may be promising targets to inhibit the metastatic properties of tumor cells.

Interestingly, some cancers express the actin reorganizing protein L-plastin, which is normally leukocyte specific and not present in non-hematopoietic cells [8,9]. After the discovery of L-plastin as a tumor-associated protein, its role as a potential tumor marker was examined [10,11]. However, results of clinical studies comparing L-plastin expression in tumor specimen from patients with disease severity were ambiguous (for review see [12]). While expression of L-plastin correlated with the invasive potential of colorectal cancer [13], there was no correlation between L-plastin expression and tumor progression in breast cancer [14] or melanoma [15]. One reason for this discrepancy may be that these studies did not consider the phosphorylation status of L-plastin.

The plastin family of actin-binding proteins consists of three isoforms, which show tissue specific expression. They exhibit a similar molecular organization containing two consecutive actin binding domains in the C-terminus, each consisting of two calponin homology (CH)-domains. This structure allows the organization of actin filaments into very tight bundles. In the N-terminal part there are two helix-loop-helix EF-hand Ca^2+^-binding motifs as well as a phosphorylation site on Ser5 (for review see [16]). Ser5 phosphorylation was shown to increase the actin bundling activity of L-plastin *in vitro* to promote its targeting to sites of actin assembly [17]. Regulation through phosphorylation of L-plastin has been described as a consequence of immune responses [18-20] as well as in response to signals triggering migration [21].

L-plastin function is important for cells of the innate as well as the adaptive immune system. We have demonstrated that L-plastin is crucial for immune synapse formation [19]. Furthermore, it regulates integrin-dependent adhesion and migration of both granulocytes [22,23] and T-cells [24]. From *in vitro* studies there were also hints that L-plastin plays a role in tumor cell motility (for review see [12,25,26]). However, so far no *in vivo* experiments existed investigating whether L-plastin plays a crucial role for tumor cell metastasis.

Therefore, in this study we systematically analyzed the *in vivo* role of L-plastin expression as well as L-plastin phosphorylation for tumor cell growth and tumor metastasis formation in a xenograft mouse model after subcutaneous or intracardial injection respectively of different human cancer cells.

## Results

### Knock-down of L-plastin in human prostate cancer cells reduces tumor growth *in vivo*

**Figure 1:**
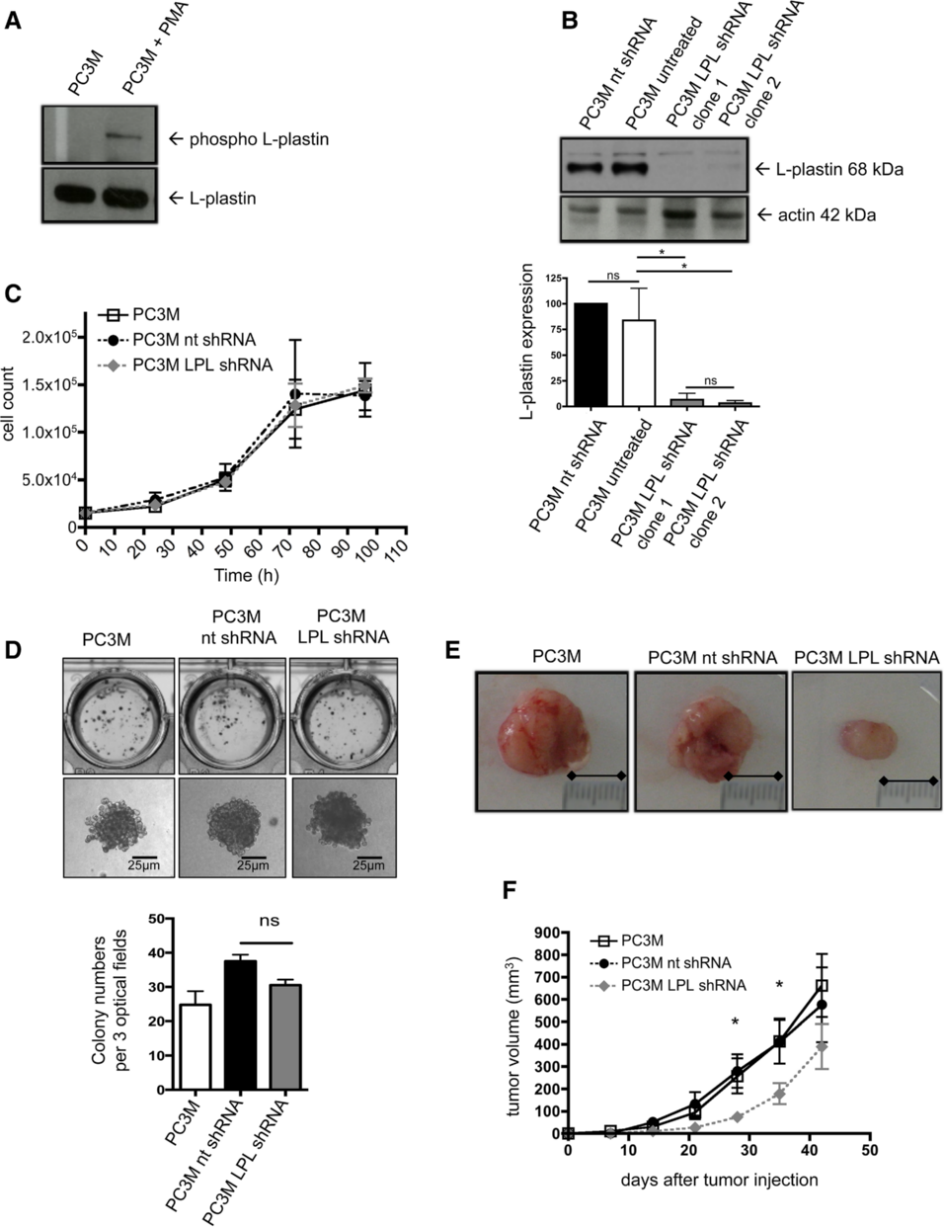
**L-plastin knock-down in prostate carcinoma cells. (A)** L-plastin is expressed in PC3M cells and gets phosphorylated following PMA stimulation. Phosphorylation of L-plastin in untreated PC3M or PC3M preincubated with 10^-8^ M PMA for 30 min was determined by Western blot with a phospho-Ser5 L-plastin specific antibody (upper panel). Total expression of L-plastin (lower panel). **(B)** ShRNA mediated downregulation of L-plastin in PC3M. PC3M infected with lentivirus encoding L-plastin (LPL) or non-targeting (nt) shRNA were analysed by Western blot for LPL protein (upper part), and β-actin as loading control. Quantification by densitometry of three independent experiments is shown in the lower part. For each experiment L-plastin levels were normalized for β-actin. The amount of L-plastin in untreated PC3M was set as 100%. Results were analyzed using one-way ANOVA (n = 3) (* = p < 0,05). **(C)** No influence of L-plastin knock-down on contact dependent in vitro proliferation of PC3M. Data represent means ± SEM, experiments were repeated at least 3 times with samples in triplicate. **(D)** Unchanged anchorage independent in vitro proliferation of L-plastin knock-down PC3M. PC3M (as indicated) were allowed to grow in soft agar for 21 days. Cell culture plates with the colonies were scanned using a transmission light scanner (upper panel). Single colonies were acquired with a 20x objective (lower panel). Bars indicate the colony numbers in three optical fields. Data represent means ± SEM. Results were analyzed using one-way ANOVA (n = 4; ns = not significant). **(E/F)** Knock-down of L-plastin in PC3M leads to reduced tumor volumes in vivo. PC3M (as indicated) were injected subcutaneously into mice. **(E)** Representative images of excised tumors at day 42. **(F)** Tumor volumes calculated weekly (n = 10; *p < 0.05 for PC3M vs. PC3M LPL shRNA and PC3M nt shRNA vs. PC3M LPL shRNA).

To investigate the effect of endogenous L-plastin expression on tumor cell behaviour, the L-plastin positive human prostate cancer cell line PC-3Mpro4/luc was used (in the following designated as PC3M). These PC3M cells, in addition, stably express luciferase and thus, exhibit luminescence in the presence of luciferin (Additional file 1: Figure S1A, upper two panels). L-plastin was phosphorylatable in these cells since PMA (phorbol 12-myristate 13-acetate) stimulation of PC3M cells led to phosphorylation of the endogenous L-plastin, as detected by an anti-phospho Ser5 L-plastin antibody (Figure 1A). To reduce L-plastin expression in PC3M cells we used a lentiviral system to deliver an L-plastin specific shRNA. In parallel a non-targeting shRNA was expressed. As illustrated in Figure 1B, treatment with L-plastin specific shRNA (LPL shRNA) resulted in an over 90% reduction in L-plastin protein expression in contrast to cells treated with non-targeting control shRNA (nt shRNA) or untreated PC3M (PC3M untreated). Note that bioluminescent activity was not influenced by shRNA transfection (Additional file 1: Figure S1A, lower two panels).

Contact dependent as well as anchorage independent cell growth was then analyzed *in vitro.* For contact dependent proliferation, cell growth on tissue culture plates was counted daily up to 96 hours (Figure 1C). The knock-down of L-plastin had no effect on proliferation in this system. Anchorage independent proliferation was determined with a soft agar assay [27]. This assay did also not unravel a growth disadvantage of PC3M cells due to a knock-down of L-plastin (Figure 1D). Together, knock-down of L-plastin had no effect on *in vitro* proliferation.

We next analyzed the *in vivo* tumor growth in a xenograft mouse model. PC3M cells either containing endogenous L-plastin, or PC3M cells expressing nt shRNA or the LPL shRNA were injected subcutaneously in the left leg of nude mice. These mice lack a thymus and are not able to induce an adaptive immune response against human cells [28]. Tumor growth was analyzed weekly over 42 days. Primary tumors were excised at day 42 and tumor volume was calculated. Surprisingly, knock-down of L-plastin reduced significantly the primary tumor growth *in vivo* (Figure 1E and F). Since the *in vitro* proliferation was not significantly changed by knock-down of L-plastin, this diminished tumor growth could be due to a malfunction in colonialization.

### Knock-down of L-plastin interferes with processes crucial for colonialization of tumor cells

**Figure 2:**
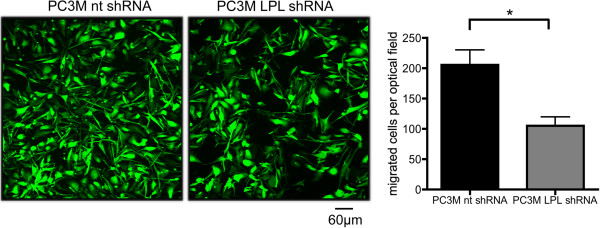
**L-plastin knock-down reduces cancer cell migration *in vitro.*** SDF1α (350 ng/ml) mediated migration towards the integrin ligand collagen I of PC3M nt shRNA and PC3M LPL shRNA cells was analyzed as described in material and methods. Cells were incubated for 18 hours for migration assays. Results were analyzed using student’s *t*-test, data represent means ± SEM (n ≥ 3; * = p < 0.05).

In order to spread and colonize adjacent or non-adjacent tissues or organs, cancer cells need to migrate through the body. To investigate whether endogenous L-plastin expression in human tumor cells facilitates this process, we first analyzed the migratory potential of PC3M cells *in vitro* in transwell assays. Tumor cell metastasis is strongly influenced by stimuli, like chemokines or integrins, surrounding the tumor cells [29]. Since L-plastin promotes integrin-mediated adhesion and migration of hematopoietic cells [16], we determined migration with the integrin ligand collagen I as a substrate and an additional chemoattractant (SDF1α) in the lower chamber of the transwell system (for details see Material and methods). Indeed, the knock-down of L-plastin in PC3M cells (PC3M LPL shRNA) significantly reduced migration (Figure 2).

### *In vivo* colonialization of human prostate cancer cells in a xenograft mouse model is reduced by knock-down of L-plastin

**Figure 3:**
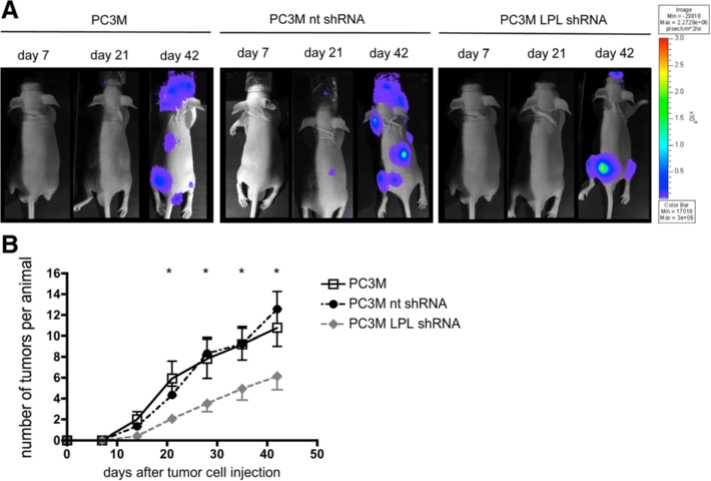
**L-plastin knock-down reduces metastasis of prostate carcinoma cells.** Tumor metastasis monitored by *in vivo* bioluminescence imaging. **(A)** Representative examples of dorsal optical images of mice injected with PC3M, PC3M nt shRNA or PC3M LPL shRNA cells at the indicated time points are shown. **(B)** Quantification of metastasis. Numbers of single spots, representing metastatic lesions, were counted from a ventral and dorsal view per animal and plotted over time after cancer cell injection. Results were analyzed using student’s *t*-test, data represent means ± SEM (n = 12 for PC3M and PC3M nt shRNA; n = 17 for PC3M LPL shRNA; * = p < 0.05 for PC3M vs. PC3M LPL shRNA and PC3M nt shRNA vs. PC3M LPL shRNA).

The experiments shown in Figure 2 suggest a function of L-plastin in the process of cancer cell spreading and induction of metastatic colonies. In the so far here used experimental model of subcutaneous tumor cell injection no spontaneous metastasis to non-adjacent organs was detected. Therefore, we switched to an established metastasis model involving intracardial injection of tumor cells in nude mice [30], to analyze whether L-plastin promotes tumor metastasis. After injection of either PC3M cells containing endogenous L-plastin or L-plastin knock-down PC3M cells (PC3 LPL shRNA) into the left cardiac ventricle of nude mice, the number of metastatic colonies was assessed by weekly ventral and dorsal bioluminescence imaging of mice and counting of luciferase-positive foci from both sides. Figure 3A shows dorsal images of day 7, 21 and 42. Indeed, L-plastin knock-down PC3M cells showed significantly reduced numbers of metastatic colonies in the animals over a period of 42 days compared to animals injected with nt shRNA transfected PC3M or untransfected PC3M cells (Figure 3A and B). These data demonstrate that L-plastin expression promotes tumor metastasis by enhancing colonialization.

### Ectopically expressed wildtype L-plastin undergoes spontaneous phosphorylation on Ser5 in melanoma cells

**Figure 4:**
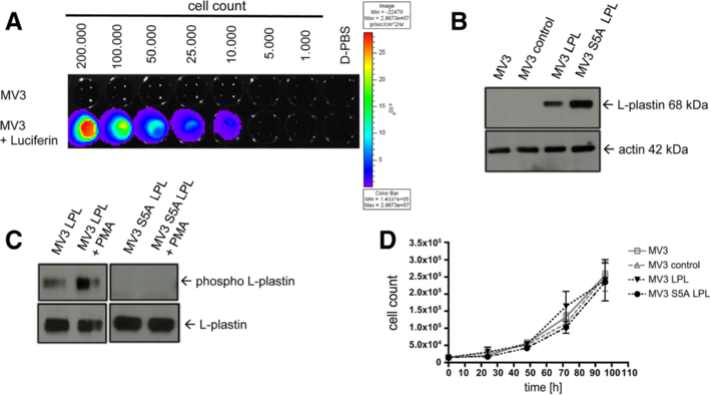
**Phosphorylation of L-plastin in melanoma cells occurs spontaneously at Ser5. (A)** Bioluminescence imaging of MV3 pCAGG3.1luc2 clone 2 (MV3) cells *in vitro*. The color bar on the right indicates the signal intensity range (p/s/cm^2^/sr). **(B)** Western blot analysis of L-plastin levels in different MV3 cells: untransfected MV3 cells (MV3), MV3 cells transfected with a control vector (MV3 control), or MV3 cells expressing either LPL (MV3 LPL) or S5A LPL (MV3 S5A LPL). **(C)** L-plastin phosphorylation, detected via a phospho Ser5 L-plastin antibody in Western Blot, occurs in MV3 cells expressing WT LPL plus and minus PMA stimulation but not in MV3 cells expressing S5A LPL. **(D)** Proliferation of the different MV3 cells *in vitro*. Data represent means ± SEM, experiments were repeated at least three times with samples in triplicates.

For an independent confirmation that L-plastin plays a role in *in vivo* colonialization of tumor cells the human melanoma cell line MV3 was used, since these cells were negative for endogenous L-plastin expression. This gives the possibility to analyze the effect of L-plastin on metastasis even in a phosphorylation-dependent manner via a transfection of wildtype or non-phosphorylatable L-plastin into these melanoma cells. To allow *in vivo* imaging of tumor cells, first, a cDNA encoding the luciferase 2 gene (pCAGG3.1-luc2) was transfected in MV3 cells and luciferase 2 expressing cells (designated as MV3) were selected, cloned and tested for their bioluminescence activity as well as for their proliferative and migratory capability. Luciferase expression had no influence on proliferation and migration of the cells (data not shown). Cells with a stable and strong bioluminescence activity (Figure 4A) were chosen for further transfections to generate stable L-plastin expressing clones.

Up to now, the exact phosphorylation site of L-plastin in melanoma cells was not determined. From our previous work we know, that a S5AS7A mutant of L-plastin prevents phosphorylation [15]. In leukocytes and Vero cells Ser5 was identified as the site of phosphorylation [17,18,22,31]. To investigate if Ser5 is also the crucial site of phosphorylation in melanoma cells, a cDNA encoding wildtype L-plastin (LPL) or a mutated version of L-plastin (S5A LPL) were transfected into MV3 cells. Immunoblot analysis of generated cell clones revealed no expression of L-plastin in untransfected cells or cells transfected with a vector control (MV3 control), but high L-plastin expression was found in MV3 LPL and MV3 S5A LPL cells (Figure 4B). Note that the bioluminescent activity was not influenced by transfection or L-plastin expression (Additional file 1: Figure S1B). Interestingly, the wildtype L-plastin is already partially phosphorylated in the absence of further external stimuli. After stimulation of these cells with the phorbol ester PMA, the amount of phosphorylated L-plastin was enhanced as observed for the PC3 cells. These findings, in addition, imply that a protein kinase C is involved in the phosphorylation of L-plastin in these tumor cells. Such an L-plastin phosphorylation was, however, only observed in wildtype L-plastin expressing cells. In contrast, S5A mutation within the L-plastin molecule prevented phosphorylation as detected with a specific anti phospho Ser5 L-plastin antibody (Figure 4C) or in an assay analyzing total L-plastin phosphorylation (Additional file 2: Figure S2). Together, these data suggest that Ser5 is the functional relevant phosphorylation site of L-plastin for melanoma cell colonialization *in vivo*. In line with the data obtained with PC3M cells, expression of L-plastin had no effect on tumor cell proliferation *in vitro* (Figure 4D).

### L-plastin phosphorylation on Ser5 is required for the promoting effect of L-plastin on tumor cell migration *in vitro*

**Figure 5:**
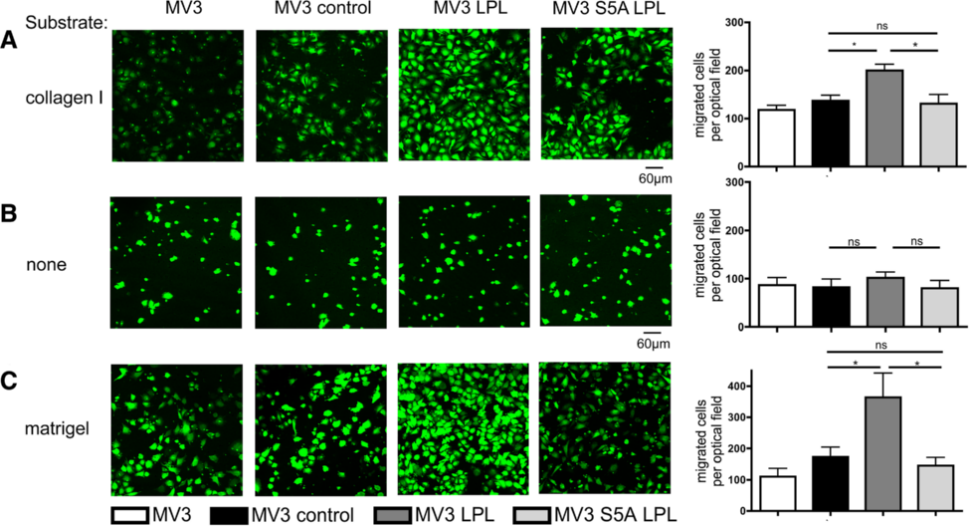
**L-plastin phosphorylation is important for migration and invasion of tumor cells *in vitro. *(A)** migration in the presence or **(B)** absence of collagen I and **(C)** invasion into matrigel of MV3, MV3 control, MV3 LPL and MV3 S5A LPL cells. Migration and invasion were analyzed using 10% FCS as chemoattractant as described in materials and methods. Cells were incubated 3 hours for migration assays and 18 hours for invasion assays. Left part: representative confocal laser scan microscopy of migrated cells. Right part: statistics of at least three independent experiments were calculated using one-way ANOVA, values represent means ± SEM (* = p < 0.05; ns = not significant).

Following the biochemical proof that mutation at Ser5 to alanine prevents phosphorylation, we next checked the functional consequences of L-plastin phosphorylation on Ser5 in melanoma cells. Thus, MV3 cells expressing either wildtype or S5A L-plastin were tested for their migratory and invasive potential *in vitro*. With collagen I as substrate (Figure 5A) transwell assays showed increased migration in the presence of wildtype L-plastin expression (MV3 LPL). This increase in the migratory capacity was abolished, if MV3 cells expressed only the non-phosphorylatable S5A LPL (Figure 5A). Chemotaxis in the absence of adhesive substrates was not influenced by either expression or phosphorylation of L-plastin, indicating that phosphorylation of Ser5 in L-plastin is important for integrin-mediated migration (Figure 5B). In a next step, invasiveness was investigated using a matrigel invasion assay [15]. Again, expression of L-plastin increased the number of transmigrating cells in contrast to cells without L-plastin expression or cells expressing the S5A LPL (Figure 5C). Thus, only expression of L-plastin phosphorylatable on Ser5 facilitated substrate-guided migration and invasiveness of MV3 cells *in vitro*.

### L-plastin enhances *in vivo* metastasis of human melanoma cells in a phosphorylation dependent manner

**Figure 6:**
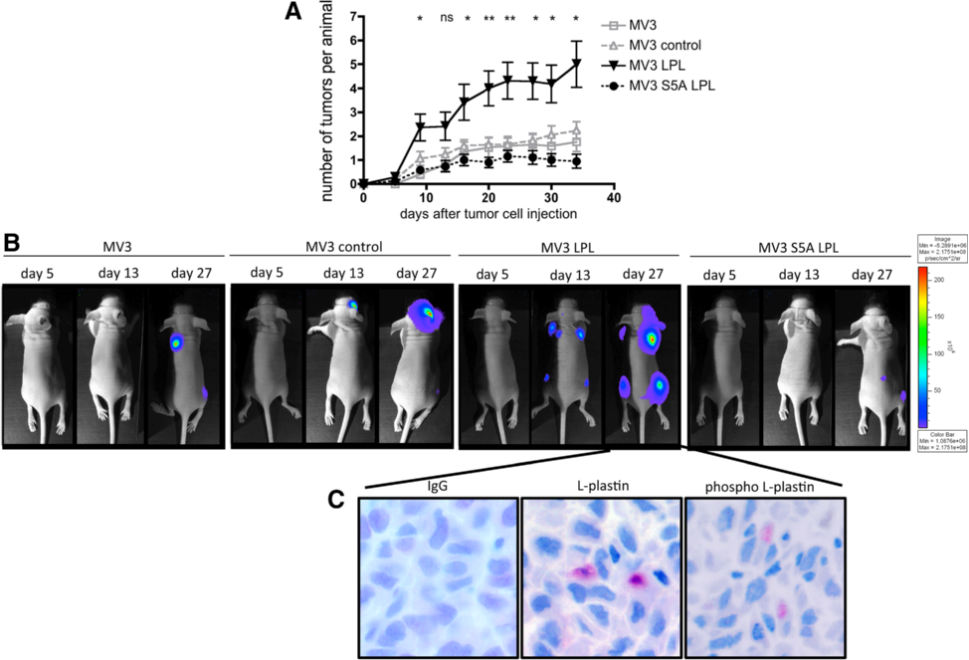
**L-plastin expression enhances metastasis in a phosphorylation dependent manner. (A)** Quantification of tumor metastasis monitored by bioluminescence imaging. Numbers of single spots, representing metastatic lesions, were counted from a ventral and dorsal view per animal and plotted over time after cancer cell injection. Results were analyzed using student’s *t*-test, data represent means ± SEM (n = 12 for MV3 and MV3 control; n = 13 for MV3 LPL and MV3 S5A LPL; * = p < 0.05 and ** = p < 0.01 for MV3 LPL vs. MV3 or MV3 control or MV3 S5A LPL; ns = not significant). **(B)** Representative pictures of dorsal optical images obtained at the indicated time points after injection of mice with MV3, MV3 control, MV3 LPL and MV3 S5A LPL cells. **(C)** L-plastin expression and phosphorylation in metastatic lesions of mice injected with MV3 LPL cells. Representative immunohistochemical pictures of excised metastatic lesions of mice bearing MV3 LPL tumors. They were stained for IgG control, total L-plastin and phospho Ser5 L-plastin.

The subsequent experiments were designed to determine whether expression of L-plastin and its phosphorylation on Ser5 are crucial not only for melanoma cell migration and invasion *in vitro*, but also for the enhancement of tumor cell metastasis *in vivo*. Indeed, expression of wildtype L-plastin in MV3 cells led to an increased number of metastatic colonies in mice after intracardial injection of the tumor cells (Figure 6A). In marked contrast, expression of the non-phosphorylatable L-plastin variant did not influence cancer cell metastasis. Figure 6B shows representative bioluminescence images on day 5, 13, 27 from all groups. To investigate whether wildtype L-plastin is still phosphorylated in the metastatic colonies *in vivo*, tumors were excised and analyzed by immunohistochemistry using the phospho L-plastin antibody. Figure 6C demonstrates that phosphorylated L-plastin can be detected in metastatic tumor cells. Together, these data demonstrate, that ectopic expression of L-plastin in tumor cells enhances metastasis and that, strikingly, not only expression, but also phosphorylation of L-plastin is important to promote tumor metastasis.

## Discussion

In order to metastasize, tumor cells must be able to leave the primary tumor and to colonize other tissues. One important step in this process is the dynamic rearrangement of the actin cytoskeleton via actin binding proteins (for review see [6]). In contrast to untransformed tissue cells, a variety of human tumor cells express the primarily leukocyte specific protein L-plastin [9]. Yet, its cellular function *in vivo* and its causal relationship to tumor cell metastasis remained unclear. Thus, contradictory results regarding the correlation of L-plastin expression with tumor progression in patients in different tumor entities were found [13-15].

Hints for a role of L-plastin expression and its phosphorylation in metastasis came from our prior experiments showing that expression of wildtype L-plastin, but not of its non-phosphorylatable counterpart S5AS7A L-plastin strongly enhanced the capacity of MV3 melanoma cells to invade into matrigel *in vitro*[15]. These studies gave rise to the assumption that (i) the expression of L-plastin and (ii) its ability to become phosphorylated may provide tumor cells an advantage to form metastases. Our aim was to test this hypothesis for human cancer cells *in vivo* by using a xenograft mouse model. We found that expression of L-plastin in tumor cells indeed promotes their metastasis in mice. This could be demonstrated in two independent experimental systems, namely either knock-down of endogenous L-plastin expression in human prostate carcinoma cells or cDNA mediated L-plastin expression in L-plastin negative melanoma cells and subsequent injection of these cells into mice. Tumor cell dissemination and metastatic colony formation was followed by bioluminescence imaging and was finally confirmed by histological analysis. Metastases occurred preferentially in bone, which is likely in part due to the injection of the tumor cells into the left ventricle of the heart resulting in arterial distribution. ShRNA induced lower expression levels of endogenous L-plastin resulted in decreased metastasis. In line with this finding ectopic expression of wildtype L-plastin raised the amount of metastatic spots in the mice. Importantly, in contrast to wildtype L-plastin, expression of non-phosphorylatable L-plastin (S5A LPL) in melanoma cells did not increase their metastatic potential. These analyses demonstrate for the first time *in vivo* that L-plastin expression combined with L-plastin phosphorylation on Ser5 promotes tumor metastasis*,* suggesting that this combination may be an important risk factor for cancer patients. Coming along with a higher number of metastatic colonies due to L-plastin expression and phosphorylation, a trend towards a prolonged survival of the animals was observed if L-plastin was not expressed, or if the non-phosphorylatable mutant (S5A LPL) was expressed in the tumor cells (data not shown).

Not just the formation of metastatic colonies after intracardial injection of tumor cells in mice was reduced, but also the primary subcutaneous tumor growth was inhibited. Considering no influence of L-plastin neither on contact dependent nor on anchorage independent proliferation of the tumor cells *in vitro*, further aspects of the process of the outgrowth of a primary tumor seem to be influenced, namely the adhesion and colonialization of the tumor cells after injection. Adhesion, which is next to the migration of tumor cells one of the main steps of colonialization, is coordinated by integrins. Mechanistically, the enhanced metastatic behaviour of wildtype L-plastin expressing cancer cells may be also mediated by avidity regulation of integrins, since only migration in the presence of integrin ligands was enhanced by expression of wildtype L-plastin. In contrast, expression of non-phosphorylatable L-plastin did not show this effect. The resulting hypothesis that phosphorylation of L-plastin is crucial for its action on integrins is supported by the fact that L-plastin-derived peptides induced activation of αvβ3-mediated adhesion of erythroleukemia cells [23] as well as by recent data showing that L-plastin expression [19] and phosphorylation [20,32] regulates the accumulation of the β2-integrin LFA-1 at the immune synapse of T-cells and antigen-presenting cells. A direct interaction of L-plastin with β-integrins was demonstrated for T-cells [19,20] as well as for breast (MCF-7) or prostate (PC3) cancer cells [33]. Notably, *in vitro*, L-plastin was found in focal adhesion sites of MV3 melanoma [15] and Vero kidney epithelial cells [34]. For the latter it was even demonstrated that L-plastin phosphorylation modulates actin dynamics in focal adhesions. Thus, L-plastin phosphorylation may be important for integrin-mediated tumor cell adhesion: Firstly, via connecting integrins to the actin cytoskeleton and thereby increasing the integrin avidity at podosomes. Secondly, via modulating the actin cytoskeleton within these adhesive structures, which is important for stabilizing these complexes. Interestingly, the activity of matrix degrading enzymes, like seprase, is also regulated by integrin clustering within invadopodia [35]. Thus, a connection between L-plastin phosphorylation and integrin avidity regulation may explain the enhanced metastatic behaviour of L-plastin expressing tumor cells observed *in vivo*, since integrins play an important role for tumor cell migration, extravasation and adhesion. The identification of the integrins involved in L-plastin mediated tumor metastasis will be the subject of further investigations. Note, however, that the behaviour of cancer cells *in vivo* is strongly influenced by the local micro-environment. Thus, the dependence of a certain tumor on L-plastin expression and phosphorylation as well as its mode of function can vary in different tumor entities.

Taken together, our systematic *in vivo* analysis demonstrates for the first time that ectopic expression of L-plastin promotes tumor metastasis. Importantly, this L-plastin needs to be phosphorylatable on Ser5. Failure to take this L-plastin phosphorylation into account in studies, aiming to find a correlation between L-plastin expression and the severity of cancer diseases, may explain the non-conclusive results found in different tumor entities of patients [13-15]. Thus, future studies are needed to establish whether L-plastin phosphorylation on Ser5 can serve as a prognostic marker. Finally, our results suggest tissue or tumor specific inhibition of L-plastin phosphorylation as a potential new therapeutic strategy to prevent metastasis of cancer. In this regard L-plastin has the advantage that, unlike other actin-binding proteins, it is not expressed in untransformed cells of the respective non-hematopoietic tissues.

## Conclusion

In summary, we show for the first time *in vivo*, via a preclinical mouse model, that ectopic expression of L-plastin in tumor cells promotes metastasis. Importantly, not the expression of L-plastin alone, but rather its additionally required phosphorylation on Ser5, enhances metastasis. Therefore, L-plastin phosphorylation on Ser5 may serve as novel prognostic marker. Given that L-plastin is selectively expressed in malignant cells – but not in untransformed non-hematopoietic cells - targeting of L-plastin expression or inhibition of L-plastin phosphorylation may open up novel therapeutic strategies to prevent tumor metastasis.

## Materials and methods

### Cell culture

The human prostate carcinoma cell line PC-3Mpro4/luc (in the following abbreviated as PC3M) (a generous gift of Dr. M. Cecchini, University of Berne, Switzerland) [36] was grown in high glucose DMEM medium supplemented with 10% fetal calf serum (FCS), w/o L-glutamine. The human melanoma cell line MV3 (a generous gift of Dr. van Muijen, University Hospital Nijmegen, The Netherlands) was grown in RPMI 1640 medium supplemented with 10% FCS, 2 mM glutamine at 37°C in an humidified atmosphere containing 5% CO_2_. Cell lines were tested by genome sequencing (DSMZ, Braunschweig, Germany).

### Generation of an anti phospho Ser5 L-plastin antiserum

Rabbit polyclonal anti-phospho L-plastin antiserum against Ser5 phosphorylated L-plastin was produced by Eurogentec according to the Speedy 28-day program. This antiserum was generated against an L-plastin peptide in which Ser5 was phosphorylated. Western blotting with the purified antiserum was performed in a dilution of 1:2000.

### Mice

BALB/c nude Mice (CAnN.Cg-*Foxn1*^*nu*^/Crl) were purchased from Charles River and were maintained in a specific pathogen-free facility of the Heidelberg University with controlled light/dark rhythm, temperature and humidity in concordance with animal care regulations. Cages, bedding, food and water were all autoclaved. All experimental procedures involving animals were approved by the responsible agency in compliance with institutional and German governmental requirements.

### Lentivirus-mediated knock-down and generation of stable knock-down cells

MISSION^®^ Lentiviral Transduction Particles, containing short hairpin (sh) RNA lentiviral vectors based on pLKO.1-puro containing a puromycin resistance gene and targeting five different sequences on the human L-plastin gene and a non-targeting shRNA vector as a control, were obtained from Sigma-Aldrich (Munich). Self-inactivating replication incompetent viral particles containing shRNAs were obtained from ImaGenes (Berlin). Infection was performed according to the manufacturer’s instructions. The shRNA used included the mature sense sequence for L-plastin: CCGGGCGGACATTTAGGAACTGGATCTCGAGATCCAGTTCCTAAATGTCCGCTTTTTG.

### Plasmids

The cDNA of wild-type (wt) full-length L-plastin and S5A-L-plastin, which were used to clone the expression plasmid vectors, were described before [15]. The entire coding region of wt full-length L-plastin cDNA (LPL) and S5A-L-plastin (S5A LPL) cDNA were amplified from L-plastin or S5A L-plastin by polymerase chain reaction (PCR) introducing a 5′and 3′*BamH*I restriction site and inserted into *BamH*I digested pSELECT-hygro-mcs vector (InvivoGen). pSELECT-hygro-mcs with an EGFP insert served as control.

The pCAGG3.1-luc2 plasmid containing the mammalian-codon optimized firefly luciferase 2 was used as described elsewhere [37]. All constructs were checked by standard DNA sequencing.

### Transfections and establishment of stable protein expression in cell populations

Plasmids expressing the firefly luciferase gene (pCAGG3.1-luc2) and plasmids expressing either wt- or S5A-L-plastin cDNA or EGFP were transfected into MV3 cells using Lipofectamine 2000 reagent (Invitrogen) according to the manufacturer’s instructions. Stable expression of transferred luciferase gene was controlled by bioluminescence imaging in complete medium supplemented with 150 μg/ml D-luciferin (Synchem) by *in vitro* imaging using the IVIS™ camera system (Xenogen, Alameda, CA, USA). Bioluminescent single cell clones were amplified in culture and tested for stable luminescence and proliferative potential *in vitro*.

Stable expression of transferred L-plastin genes was checked by immunoblots. L-plastin expressing single cell clones were amplified in culture and characterized for stable luminescence levels, L-plastin expression as well as migration *in vitro.* Positive MV3 LPL or MV3 S5A LPL clones, respectively, were expanded and initially selected and used for further *in vitro* and *in vivo* studies.

### SDS-Polyacrylamide gel electrophoresis followed by Western-blotting

Protein lysates from cells were obtained as previously described [15]. Cells were washed once in ice-cold Tris-buffered saline (TBS), lysed for 30 minutes in TKM-buffer (50 mM Tris–HCl pH 7.5, 1% NP40, 25 mM KCl, 5 mM MgCl_2_, 1 mM NaVO_4_, 5 mM NaF, 20 μg/ml each Leupeptin/Aprotinin) and nuclei were removed by centrifugation. SDS-polyacrylamide gel electrophoresis (SDS-PAGE) was performed according to standard procedures. Proteins were blotted onto polyvinylidene difluoride (PVDF, Immobilon-P, Merck-Millipore, Darmstadt, Germany) membranes. Membranes were blocked with 10% BSA in PBS. For protein detection the membranes were incubated with primary antibodies for L-plastin (clone LPL4A.1, Neomarkers) (1 μg/ml), phospho L-plastin (our own antibody) or ß-actin (clone A2066, Sigma-Aldrich) (1:900) overnight at 4°C, followed by incubation with horseradish peroxidase-conjugated anti-rabbit or anti-mouse secondary antibodies (Jackson ImmunoResearch). Proteins were visualized with ECL (Amersham-Pharmacia). Densitometric analysis was performed using a GS-800-densitometer and “Quantitiy One” software (BioRad). L-plastin phosphorylation was calculated as the ratio between phospho-protein and total protein. Data from densitometric analysis are reported as mean ± SEM.

### *In vitro* cell proliferation and soft agar assays

For contact dependent proliferation, cells were washed, trypsinized and adjusted to a density of 1.5 × 10^4^ cells/ml in culture medium. Cells in 1 ml aliquots were seeded to grow in 24-well plastic plates. Cell numbers were counted at the times indicated using a Neubauer chamber.

The soft agar assays (anchorage independent proliferation) were performed by preparing a 0.5% base agar and a 0.35% top agar with RPMI1640 and FCS (10% v/v final concentration). The layer of 0.5% agar was poured in 24 well plates. PC3M, PC3M nt shRNA or PC3M LPL shRNA cells were resuspended in 0.35% top agar at a density of 1.5 × 10^5^ cells/ml. Cell suspensions were poured on the top of the base layer. After solidification the agar was overlaid with RPMI1640 plus 10% FCS and plates were incubated at 37°C in the presence of 5% CO_2_. The colonies were counted in 3 optical fields in each sample at day 13.

### *In vitro* migration assay

*In vitro* xcell migration was analyzed essentially as previously described [15], using transwell migration inserts (PET membrane, 8 μm pore size, 6 mm diameter, BD Biosciences) coated with 20 μg/ml collagen I in 0.02 N acetic acid at the bottom side and containing soluble chemoattractants (600 μl of either RPMI 1640 medium containing SDF1α (350 ng/ml) for PC3M cells, or RPMI 1640 supplemented with 10% FCS for MV3 cells) in the lower compartment. Cells were labeled for 15 minutes at 37°C/5% CO_2_ with 1 μM CFDA-SE (molecular probes) in PBS prior to performing the migration assay. 2.5 × 10^5^ cells/ml for MV3 and 1.5 × 10^5^ cells/ml for PC3M were allowed to transmigrate, then filters were removed and cells from the upper membrane surface were wiped off with a cotton swab. Filters were then washed, fixed and mounted on glass slides. Cells that had migrated to the coated lower side of the filter were detected by confocal laser scan microscopy and cells in four defined optical fields were counted for each filter. Time points are indicated in the figure legends.

### *In vitro* invasion assay

To investigate the invasiveness of tumor cells BD BioCoat Matrigel invasion chambers were used. 200 μl of a cell suspension at a concentration of 1 × 10^5^ cells/ml was added to the upper compartment and cells were allowed to migrate to the lower compartment with 10% of FCS as a chemoattractant. Invasive cells were counted as mentioned above.

### Subcutaneous injection of tumor cells

5 week old male BALB/c nude mice were subcutaneously injected with 1.5 × 10^5^ PC3M cells in 100 μl D-PBS in the hind leg. Subcutaneous tumor growth was determined weekly via caliper. Tumor volume was calculated by the formula: length × width^2^ × 0.5.

### Intracardial injection of tumor cells

5 week old nude mice were anesthetized by intraperitoneal injection of 120 mg/kg ketamine hydrochloride with 16 mg/kg xylazine on the day of injection. Male animals were used for the injection of PC3M cells, female mice were used for the injection of MV3 cells. On day 0, anesthetized animals were injected with 1.5 × 10^5^ PC3M cells, or 1 × 10^5^ MV3 cells respectively, diluted in 100 μl D-PBS into the left cardiac ventricle. Anesthetized animals were placed in the IVIS100 Imaging System (Xenogen, Alameda, CA, USA) within 60 minutes after injection and imaged dorsally and ventrally 5 min after intraperitoneal injection of D-luciferin. A successful intracardial injection was indicated on day 0 by images showing systemic bioluminescence distributed throughout the body of the mouse. Only animals with evidence of a satisfactory injection continued the experiment. Assessment of subsequent metastasis was monitored *in vivo* once or twice a week, depending on the tumor model, for up to 42 days.

### Bioluminescence imaging

Bioluminescence imaging was performed with a CCD camera in a light-tight specimen box (IVIS100, Xenogen). Quantification of signals was performed by the acquisition and analysis software Living Image^®^ (Xenogen). For *in vitro* imaging, bioluminescent cells were serially diluted from 1 × 10^3^ to 2 × 10^5^ cells into black 96-well Plates. 600 μg/ml D-luciferin was added to each well 5 min before imaging. Imaging time was 1 min/plate, Binning M. For *in vivo* imaging, animals were anesthetized with isoflurane and were given D-luciferin (15 mg/ml in D-PBS; 100 μl/10 g body weight) by intraperitoneal injection. Mice were placed into the light-tight camera box with continuous exposure to isoflurane. Imaging time ranged from 10 s to 1 min, depending on the tumor model, 5 min after D-luciferin injection. Two mice were imaged each time from a dorsal and a ventral view. The photons emitted from the bioluminescent tumors or cells were detected by the IVIS100 camera system, integrated, digitized and displayed. Numbers of tumors per animal were counted by eye from a dorsal and ventral view with particular regard to the singular count of one metastatic lesion.

### Immunohistochemistry

The following primary antibodies were used: Rabbit polyclonal anti-phospho L-plastin antiserum (phospho L-plastin) and mouse monoclonal anti-L-plastin antibody (LPL4A.1). Isotype- and concentration matched control antibodies (Dako, Hamburg, Germany) served as negative controls.

Immunoenzyme staining was performed on 2-μm sections of formalin fixed, paraffin embedded murine organs. Heat induced antigen-retrieval was achieved by incubating the slides in a steam cooker for 5 minutes in citrate buffer, pH 6.1. Slides were further processed using the standard avidin-biotin-complex anti-alkaline phosphatase procedure (Vectorlabs, Burlingame, CA, USA) according to the manufacturer’s instructions. The primary antibody was added for 1 hour at 37°C. A donkey anti-rabbit biotinylated antibody, 1/100 (Dako), was used as a secondary reagent (30 min at room temperature). Naphthol AS-biphosphate (Sigma, St. Louis, MO, USA) with New-fuchsin (Merck, Darmstadt, Germany) served as the substrate for alkaline phosphatase.

### Statistical methods

Statistical analyses were performed with GraphPad Prism 4.0. Data are shown as means ± SEM. Comparisons of means were performed with two-tailed students *t*-test or one-way ANOVA test, respectively. P values were considered significant when p < 0.05.

## Competing interests

The authors declare that they have no competing interests.

## Authors’ contributions

SMR and GHW participated in study design, performing experiments, data analysis and drafting of manuscript. HK, BJ and BS supported *in vitro* experiments and cloning experiments and provided technical assistance. GVDH and GVDP provided material. FL performed immunhistochemistry experiments. GH and IN were involved in conception of study and manuscript drafting. YS participated in study design, data analysis and writing of the manuscript. All authors read and approved the manuscript.

## Supplementary Material

Additional file 1: Figure S1 Bioluminescent activity of tumor cells was not influenced by transfection of the cells. (A) Bioluminescence imaging of PC-3Mpro4/luc (PC3M) cells *in vitro*. (B) Bioluminescence imaging of MV3 cells *in vitro*. The color bar at the right indicates the signal intensity range (p/s/cm^2^/sr). Click here for file

Additional file 2: Figure S2 S5A-LPL is not phosphorylated in PMA treated MV3 cells as detected by native gel electrophoresis. A) LPL phosphorylation can be detected by native gel electrophoresis followed by Western blotting. LPL is known to be phosphorylated following PMA stimulation of T-cells [18]. This phosphorylation can be visulized by resolving proteins of untreated or PMA-treated T-cells (10^-8^ M; 30 min) on native PAGE and staining of L-plastin on the corresponding Western blot with L-plastin antibodies. In contrast to the situation with control cells, PMA-treatment leads to the occurrence of a second band. This band disappears if lysates from PMA stimulated cells were treated with alkaline phosphatase (AP). B) For an unbiased analysis of LPL phosphorylation in MV3 cells, lysates of control or PMA-treated MV3 cells expressing either wt LPL or S5A LPL were subjected to native gel electrophoresis as described in A. Only wt LPL, but not S5A-LPL showed a band shift after PMA treatment of the cells, which demonstrates that S5A-LPL is not phosphorylated. Click here for file
