# Supplementary figures and images for: Metastasis of prostate cancer and melanoma cells in a preclinical in vivo mouse model is enhanced by L-plastin expression and phosphorylation
Source: Mol Cancer. 2014 Jan 18;13:10. doi: 10.1186/1476-4598-13-10 (PMC3899628; doi:10.1186/1476-4598-13-10)

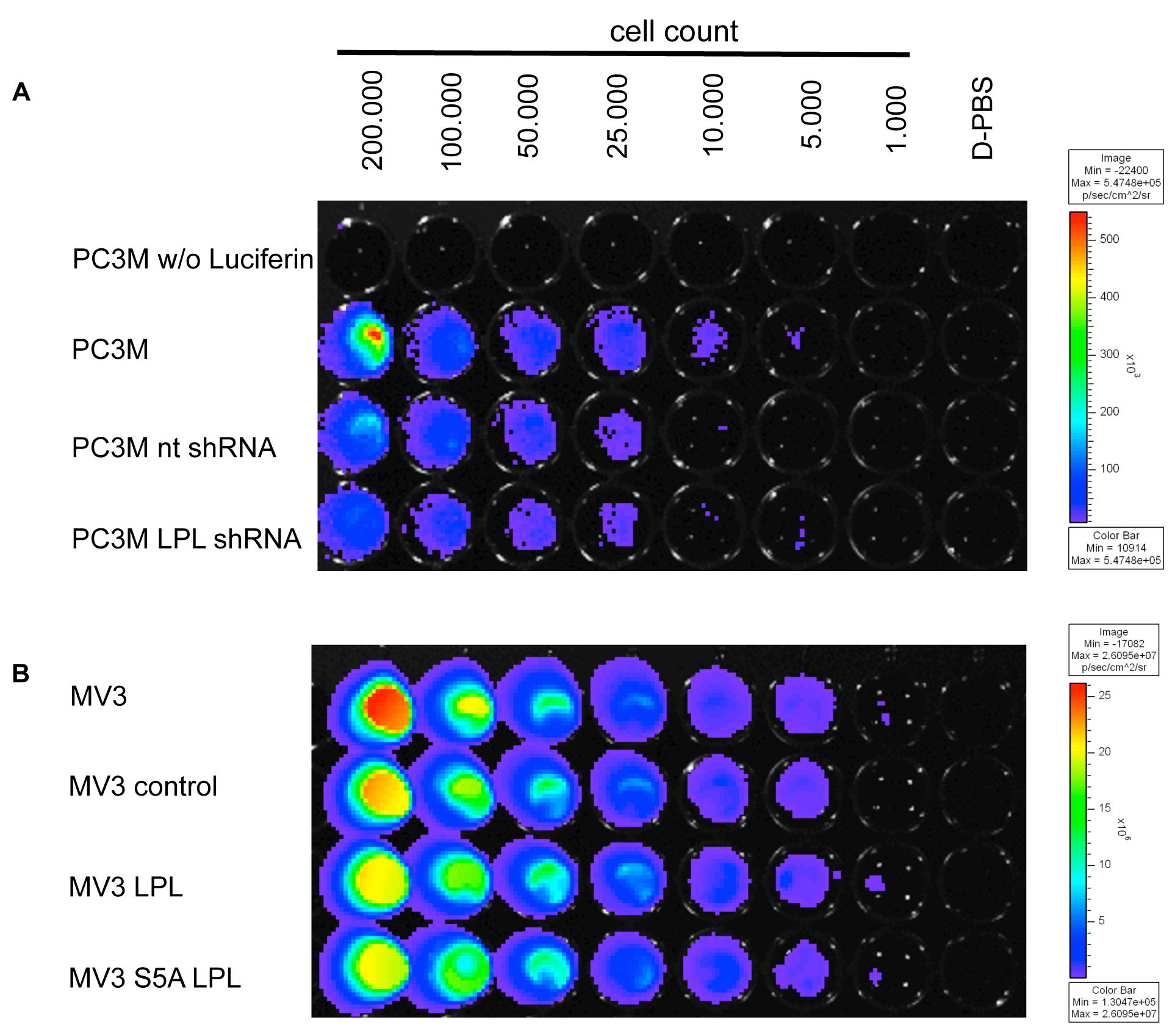

Supplement: Additional file 1: Figure S1 — Bioluminescent activity of tumor cells was not influenced by transfection of the cells. (A) Bioluminescence imaging of PC-3Mpro4/luc (PC3M) cells in vitro. (B) Bioluminescence imaging of MV3 cells in vitro. The color bar at the right indicates the signal intensity range (p/s/cm2/sr). [file 1476-4598-13-10-S1.tiff]

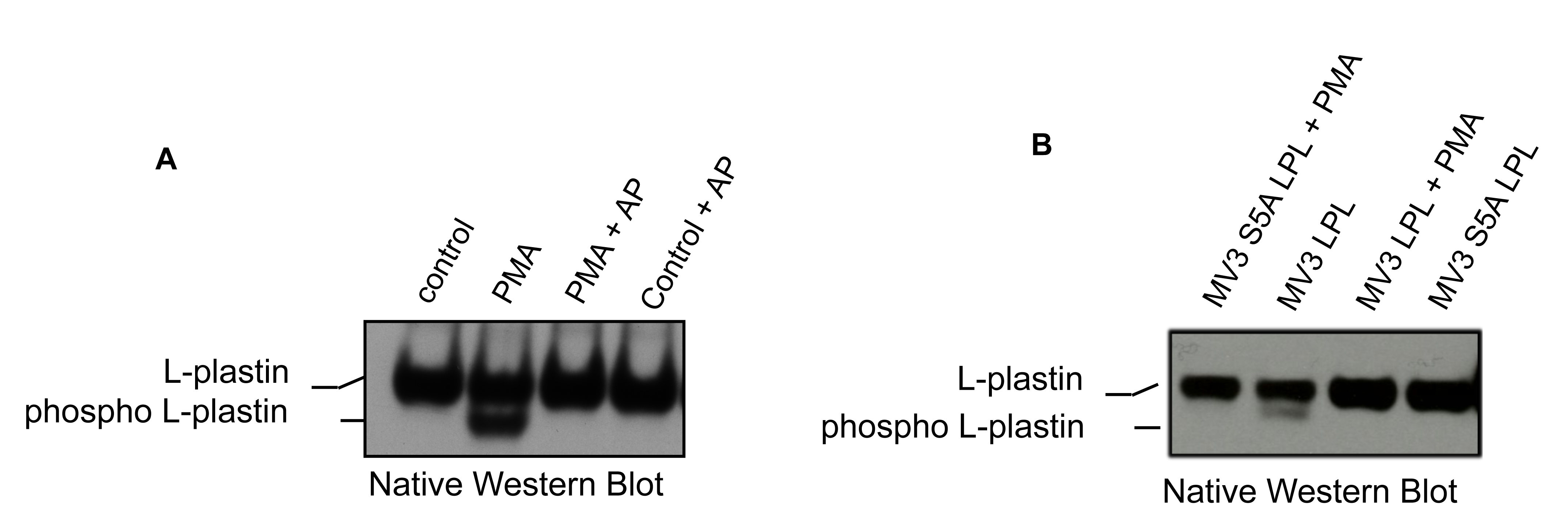

Supplement: Additional file 2: Figure S2 — S5A-LPL is not phosphorylated in PMA treated MV3 cells as detected by native gel electrophoresis. A) LPL phosphorylation can be detected by native gel electrophoresis followed by Western blotting. LPL is known to be phosphorylated following PMA stimulation of T-cells [18]. This phosphorylation can be visulized by resolving proteins of untreated or PMA-treated T-cells (10-8 M; 30 min) on native PAGE and staining of L-plastin on the corresponding Western blot with L-plastin antibodies. In contrast to the situation with control cells, PMA-treatment leads to the occurrence of a second band. This band disappears if lysates from PMA stimulated cells were treated with alkaline phosphatase (AP). B) For an unbiased analysis of LPL phosphorylation in MV3 cells, lysates of control or PMA-treated MV3 cells expressing either wt LPL or S5A LPL were subjected to native gel electrophoresis as described in A. Only wt LPL, but not S5A-LPL showed a band shift after PMA treatment of the cells, which demonstrates that S5A-LPL is not phosphorylated. [file 1476-4598-13-10-S2.tif]
